# Anomalous drainage of inferior vena cava into left atrium associated with ostium secundum atrial septal defect: a case report

**DOI:** 10.1093/ehjcr/ytae293

**Published:** 2024-07-09

**Authors:** Sanjeev Asotra, Ritesh Kumar, Praveen Dhaulta, Mansi Verma

**Affiliations:** Department of Cardiology, IGMC and Hospital Shimla, Room No. 310, 3rd floor, 171001, India; Department of Cardiology, IGMC and Hospital Shimla, Room No. 310, 3rd floor, 171001, India; Department of CTVS, IGMC and Hospital Shimla, 171001, India; Department of Radiology, IGMC and Hospital Shimla, 171001, India

**Keywords:** Case report, Anomalous drainage, Atrial septal defect, Inferior vena cava, Left atrium

## Abstract

**Background:**

Anomalous drainage of inferior vena cava (IVC) into left atrium (LA) is a rare aetiology of cyanosis in adults. This condition may be associated with atrial septal defects, anomalous pulmonary venous drainage, and pulmonary arteriovenous fistulas. This case report presents an instance of anomalous drainage of IVC into LA, associated with ostium secundum atrial septal defect (ASD). It contributes to the existing literature by highlighting the diagnostic challenges associated with this anomaly, particularly during surgical intervention.

**Case summary:**

A 38-year-old male presented with a six-year history of exertional dyspnoea and episodic palpitation. Transthoracic echocardiography revealed a large secundum (ASD), the IVC draining into LA, a left superior vena cava, and mild mitral regurgitation. These findings were further confirmed by right and left heart catheterization and CT angiogram. The patient was referred to CTVS department for surgical correction. The post-operative course was uneventful. At a 1.4-year follow-up, the patient reported significant improvement in symptoms.

**Discussion:**

We present a case of anomalous drainage of IVC into LA, associated with ostium secundum ASD. An ASD co-occurs with this condition in ∼70% of the reported cases. This anomaly differs from a low or inferior vena caval secundum ASD, where a prominent Eustachian valve can cause blood shunting from the IVC to LA. If the surgeon is not vigilant, this can be mistaken for the inferior ASD rim, potentially leading to iatrogenic diversion of IVC blood to LA upon ASD closure, resulting in cyanosis. This case underscores the diagnostic and surgical challenges associated with this condition.

**Learning points**

- This entity is different from a low or inferior vena caval secundum atrial septal defect (ASD), where a prominent Eustachian valve can result in shunting of blood from the inferior vena cava (IVC) to the left atrium (LA).
- If the surgeon is not careful, this can be mistaken for the inferior ASD rim, and he may iatrogenically divert IVC blood to the LA upon ASD closure, causing cyanosis.
- Establishing diagnosis can be difficult as most reported cases in literature were diagnosed in adulthood.
- Many imaging modalities such as transthoracic echocardiography, transoesophageal echocardiography, contrast-enhanced computed tomography, and MRI can be used to establish diagnosis nowadays.

## Introduction

The anomalous drainage of inferior vena cava (IVC) into left atrium (LA) represents a rare aetiology of cyanosis observed in adult patients.^1^ This anomaly may be associated with atrial septal defects, anomalous pulmonary venous drainage, and pulmonary arteriovenous fistulas.^2^ In exceptional cases, this anomaly may also coincide with ostium secundum atrial septal defect (ASD), coronary sinus atrial septal defect, and left superior vena cava (SVC) abnormalities. However, the literature reports only a scant number of such occurrences.^3–8^ Consequently, the natural clinical progression, clinical manifestation, and diagnostic methodologies remain inadequately defined. Surgical intervention stands as the solitary recourse for rectifying this disorder.

## Summary figure

**Table:** 

Timelines	
October 2022	Presented to hospital, echocardiography done
November 2022	CECT and cath. study done and CTVS opinion for surgery taken
2 December 2022	Surgery done
9 December 2022	Discharge from hospital
March 2023	Follow-up: 90 days; he had resumed work
2 February 2024	Follow-up: 1.2 years follow-up; doing better

## Case presentation

A 38-year-old male presented with a six-year history of exertional dyspnoea and episodic palpitation. Upon general physical examination blood pressure and heart rate were within normal limits. Cardiac auscultation revealed an ejection systolic murmur at left upper sternum and fixed split of second heart sound. Cyanosis and grade I clubbing were observed. Room air saturation, measured by pulse oximetry, was 90%. Laboratory investigations indicated haemoglobin of 17.8 g and haematocrit 60%. Chest X-ray revealed cardiomegaly with dilation of right atrium (RA) and right ventricle, as well as a dilated main pulmonary artery. Electrocardiogram showed normal sinus rhythm, P pulmonale, right ventricular enlargement, and right axis deviation.

Transthoracic echocardiography (TTE) revealed a significant secundum (ASD), along with the IVC draining into LA with LSVC with mild mitral regurgitation. As IVC was not draining into RA, saline agitation test was conducted. Initially, saline with small micro bubble was injected via right brachial vein, promptly appearing with in RA. Subsequently, saline injection through femoral vein revealed bubbles first appearing with in the LA and before traversing into RA through ASD. These findings were further confirmed by right and left heart catheterization procedure. Right femoral vein access was taken and 6F JR–IVC-LA-PVs; then, right brachiocephalic vein to SVC and RA. Left heart catheterization was subsequently carried out by right femoral artery route.

**Figure 1:**
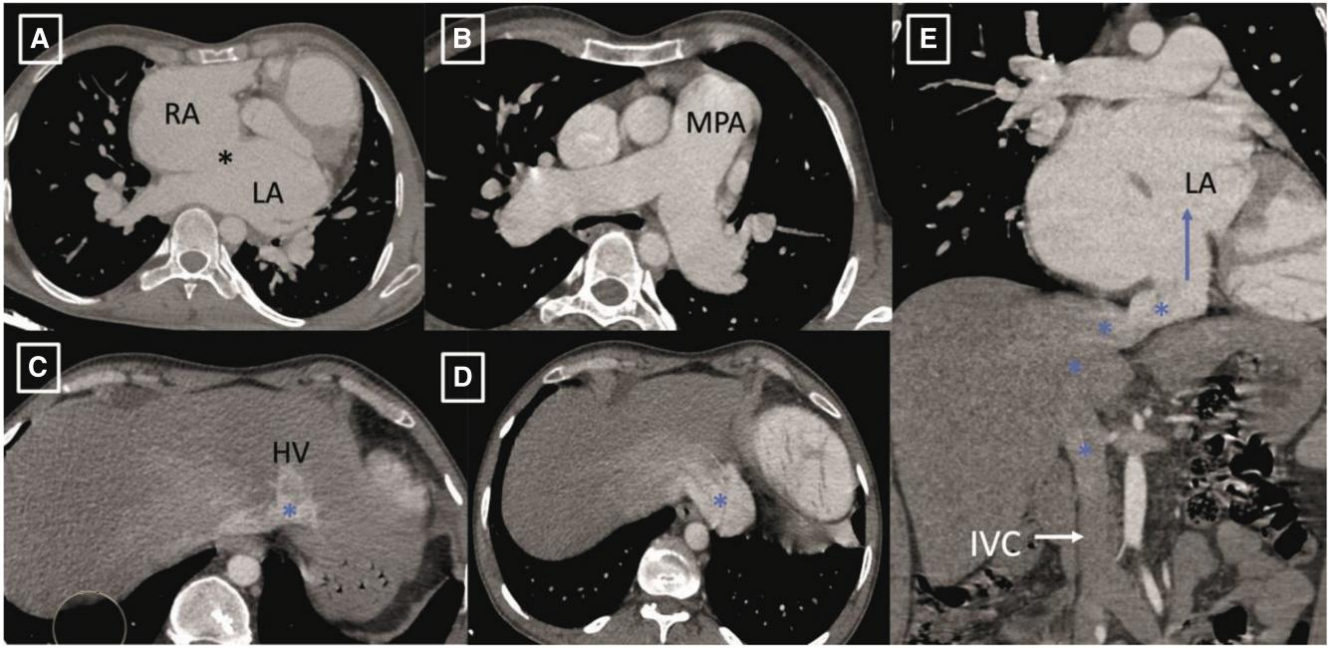
Axial computed tomography image (*A* and *B*) reveals a large ostium secundum atrial septal defect (marked by asterisk) with dilated main pulmonary artery (MPA). Axial image (*C* and *D*) depicts drainage of hepatic veins into intrahepatic portion of inferior vena cava (IVC) (marked by blue asterisk) with midline crossing of IVC to left side. Coronal image (*E*) demonstrating the course of IVC with midline crossing to left side and subsequent drainage into the left atrium (depicted by blue asterisk and blue arrow).

**Figure 2:**
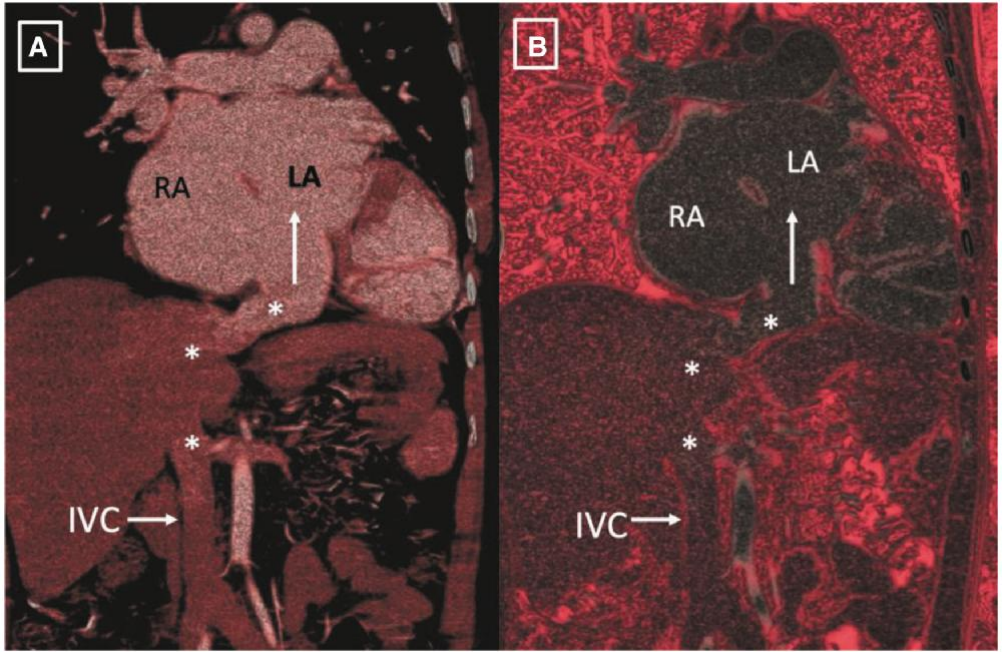
Volume rendered image (*A*) and endoluminal view (*B*) depicting the course of IVC draining into the left atrium (shown by white asterisk and white arrow).

**Figure 3:**
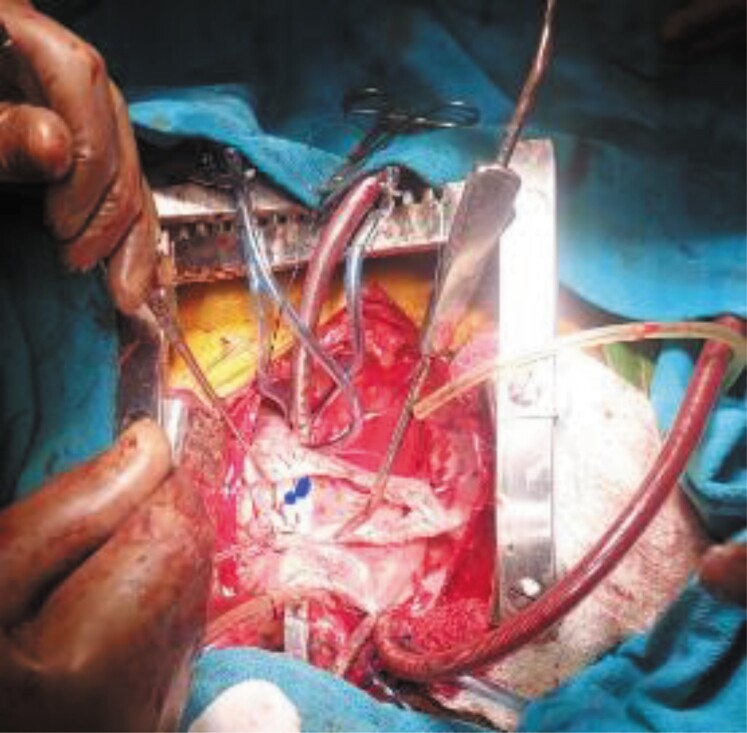
Showing IVC draining into LA (blue dot LA, line IVC).

**Figure 4:**
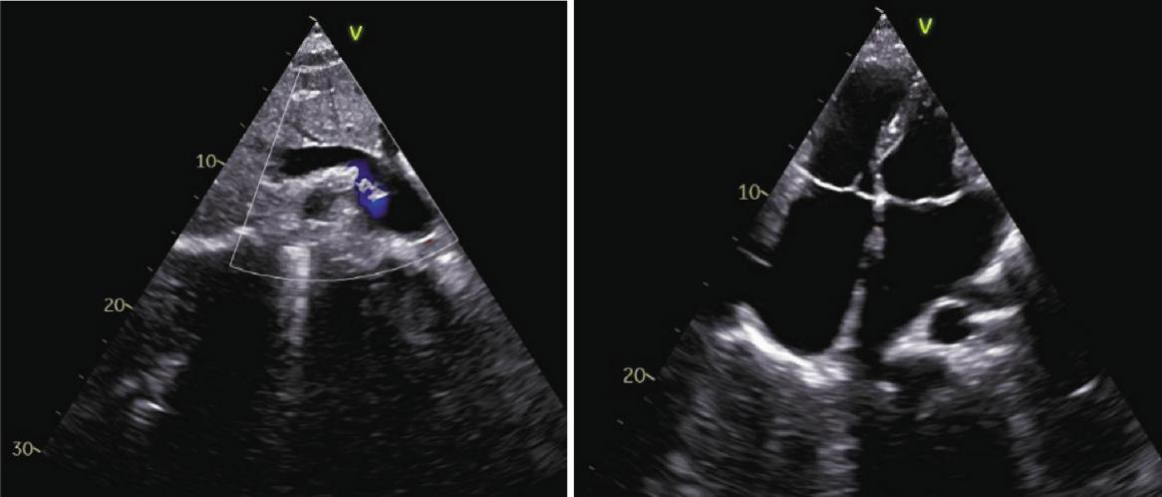
Showing corrected anatomy after surgery, routing of IVC into RA and secundum ASD, closed by Dacron patch.

The CT angiogram clearly delineated drainage of IVC into LA and large ASD and LSVC as shown in *Figures 1* and *2*. Subsequently, the patient was referred to Cardiothoracic and Vascular Surgery (CTVS) department for surgical intervention. The procedure was conducted via a midline sternotomy approach. Cannulation was directly performed on the ascending aorta and both vena cavae, with particular attention given to accessing the IVC as low as feasible. Cardiopulmonary bypass was initiated under mild hypothermia (32.8°C). The heart was arrested using antegrade cold blood cardioplegia. Upon opening RA, common atrium was observed with IVC directly draining into the LA as shown in *Figure 3*, and normal positioning and drainage of pulmonary veins. The defect was successfully closed using a Dacron patch of size 5.0 cm × 3.5 cm, with rerouting of IVC into RA. After weaning of cardiopulmonary bypass, peripheral oxygen saturation increased to 100%. The patient underwent a successful surgical correction and the post-operative recovery proceeded without complication. Corrected anatomy after surgery is shown in *Figure 4*. A follow-up examination conducted 1.2 years post-operative revealed favourable progress in patient condition.

## Discussion

The anomalous drainage of the IVC into the LA represents a rare but significant aetiology of cyanosis in the adult population.^1^ Notably, ∼70% of reported cases of this anomaly are concomitant with an ASD. Despite its rarity, a myriad of variations in the developmental pattern of the IVC have been documented. Specifically, the phenomenon of IVC drainage into the LA is an infrequent congenital anomaly, with its association with an ASD being less common. The majority of literature on this subject consists of case reports.^3–8^

This aberration may manifest in conjunction with other anomalies, including anomalous pulmonary venous drainage and pulmonary arteriovenous fistulae.^2^ The first documentation of this condition dates back to 1955 when Gardner reported it as an autopsy finding.^1^ It is hypothesized that the persistence of the right valve of the sinus venosus during embryological development underlies this abnormality. Embryonically, the sinus venosus receives venous contributions from the cardinal, umbilical, and vitelline veins. It communicates with the primitive atrium via an orifice characterized by right and left valves associated with its respective right and left horns. Ordinarily, the sinus venosus undergoes a migratory process towards the right, with subsequent disappearance of the left valve. Consequently, the left horn transforms into the coronary sinus. The right valve typically undergoes regression, eventually giving rise to structures such as the crista terminalis, Eustachian valve, and Thebesian valve. However, if the right sinus venosus valve persists and merges with the superior aspect of the secundum septum, anomalous drainage of the IVC into the LA ensues.^2,9,10^

This condition stands apart from a low or inferior vena caval secundum ASD, wherein a prominent Eustachian valve can lead to blood shunting from the IVC to the LA. Care must be exercised during surgical intervention to avoid mistaking this anomaly for the inferior ASD rim, which could inadvertently redirect IVC blood flow to the LA upon ASD closure, resulting in cyanosis. Establishing a diagnosis can pose challenges, particularly as most reported cases in the literature are diagnosed in adulthood. Various imaging modalities, including TTE, transoesophageal echocardiography, contrast-enhanced computed tomography (CECT), and magnetic resonance imaging (MRI), are now available to aid in diagnosis. Studies by Munet *et al*. utilized perfusion pulmonary scintigraphy and contrast echocardiography, while Burri *et al*. employed MRI to confirm the diagnosis. Thus, a multi-modal imaging approach, incorporating echocardiography, angiography, MRI, or CT, is often necessary to firmly establish the diagnosis. Upon confirmation of the diagnosis, surgical referral becomes imperative, as this remains the sole therapeutic option available.

## Lead author biography


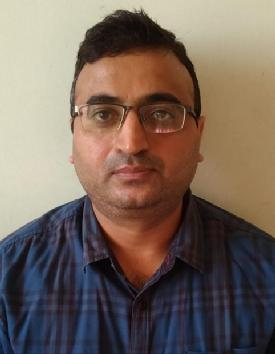


Dr Ritesh is presently working as assistant professor in Department of Cardiology at IGMC Shimla. He has interest in intervention cardiology. He is very hardworking.

##  

**Consent:** The authors confirm that a written consent for submission and publication of this case report including images and associated text has been obtained from patient in the line with COPE guidance.

**Funding:** Nothing to declare.

## Data Availability

All data related to this case report are presented in the published manuscript.
